# Optimal Item Calibration in the Context of the Swedish Scholastic Aptitude Test

**DOI:** 10.1177/01466216261420758

**Published:** 2026-02-06

**Authors:** Jonas Bjermo, Ellinor Fackle Fornius, Frank Miller

**Affiliations:** 1 Department of Computer and Information Science, 4566 Linköping University, Linköping, Sweden; 2 Department of Statistics, 7675 Stockholm University, Stockholm, Sweden

**Keywords:** item response theory, optimal design, 3PL model, simulation study, SweSAT

## Abstract

Large-scale achievement tests require the existence of item banks with items for use in future tests. Before an item is included into the bank, its characteristics need to be estimated. The process of estimating the item characteristics is called item calibration. For the quality of the future achievement tests, it is important to perform this calibration well and it is desirable to estimate the item characteristics as efficiently as possible. Methods of optimal design have been developed to allocate pretest items to examinees with the most suited ability. Theoretical evidence shows advantages with using ability-dependent allocation of pretest items. However, it is not clear whether these theoretical results hold also in a real testing situation. In this paper, we investigate the performance of an optimal ability-dependent allocation in the context of the Swedish Scholastic Aptitude Test (SweSAT) and quantify the gain from using the optimal allocation. On average over all items, we see an improved precision of calibration. While this average improvement is moderate, we are able to identify for what kind of items the method works well. This enables targeting specific item types for optimal calibration. We also discuss possibilities for improvements of the method.

## Introduction

Item calibration is the process of estimating the characteristics of new pretest items. The goal of item calibration is to develop a bank of items with precisely estimated item parameters ready for use in operational tests. It is of great importance that the item parameters are estimated as precisely as possible, since it directly affects the accuracy and estimated standard errors of the latent ability estimates (Cheng & Yuan, 2010). As Ali and Chang (2014) point out, well calibrated items are particularly important for computerized adaptive testing (CAT), when test items are assigned to the examinees adaptively based on gradually updated estimates of their latent ability. In this setting it is assumed that the item parameters have been estimated with enough precision to be treated as the true ones (van der Linden & Pashley, 2000).

Methods of optimal experimental design can be applied in this context to determine which examinees to select from a population such that the item parameters are estimated as efficiently as possible. The precision of the item-parameter estimates depends on the ability levels of the examinees that respond to the item. The design problem here is the problem of selecting a sample of examinees with the most suitable abilities, that is to find an optimal sampling design (Berger, 1991; Buyske, 2005). In addition to being more cost-effective, using an optimal sampling design means that the item assignment will be targeted to examinees in a better way in terms of item difficulty compared to when assigning items randomly. Assigning items with a fitting difficulty level reduces the burden of the examinee taking the test and the risk of identification of the pretest items.

Which sampling design strategy that is feasible depends on whether the calibration is conducted in an “online” or “offline” setting and whether the pretest items can be administered adaptively. In an online setting, pretest items are integrated in a test together with the operational items (Stocking, 1988). This allows items to be assigned to the examinees in an adaptive way, where the rule of the assignment is determined by an optimal design. Alternatively, the calibration can be done in a separate test, consisting of pretest items only, a so-called offline calibration (He et al., 2020).

The online calibration setting is well suited for adaptive optimal design schemes adjusted to sequentially arriving examinees, sequential calibration methods are implemented in different ways by van der Linden and Pashley (2000), Chang and Lu (2010), Ali and Chang (2014), Lu (2014) and van der Linden and Ren (2015). Zheng and Chang (2017) compared five methods for calibration in an online sequential setup. While the considered methods that are based on an optimality criterion in theory should improve parameter precision, they observed in their simulation study that a random design in many situations achieved a comparable performance.

However, not all testing situations have sequentially arriving examinees. Instead, the test is given to a large number of examinees taking the test simultaneously in parallel. Such a parallel settings is common for large-scale achievement tests, for example, for the Programme for International Student Assessment (PISA; OECD, 2018) and for the Swedish Scholastic Aptitude Test (SweSAT; Umeå University, 2021) considered in this paper. For item calibration under such parallel test settings, other optimal design methods are needed. The method for calibrating items based on an optimal design algorithm by Ul Hassan and Miller (2021) is suitable for calibration of items in a parallel setting. The method is designed for allocating a large group of examinees in parallel to items based on examinee ability. The algorithm utilizes a so-called optimal restricted design. The optimal restricted design creates a set of non-overlapping ability intervals that dictates which examinee should be given which pretest item. Once the ability of an examinee is estimated, it is known which ability interval the examinee belongs to, and the designated item can be allocated to the examinee.

The computations involved in the optimal design algorithm are based on some approximations which means the demonstrated theoretical efficiency gains are not guaranteed to hold in practice. The optimal design therefore needs to be evaluated in a real testing situation. Moreover, Zheng and Chang (2017) demonstrate that a random assignment can give comparable results as optimal designs when they evaluated several different test conditions.

In this study, we evaluate the optimal allocation strategy proposed in Ul Hassan and Miller (2021) and compare the results to the random allocation strategy. We use real data from the Swedish Scholastic Aptitude Test (SweSAT) to conduct an empirically based simulation study, designed to replicate the real calibration setting of SweSAT. Moreover, we consider a set of different simulation scenarios with varying degree of assumptions made. In this way, we are able to separate the effect of each assumption. The responses in the test round of 2018 are used to estimate item parameters that are utilized in the optimal design algorithm to decide the optimal allocation on the basis of estimated examinee ability. The calibration procedure of SweSAT is that each examinee is given the same fixed number of pretest items, in contrast to alternative schemes where the items may be given to the examinees until the standard errors of the estimates meet some criteria, or by letting a fixed number of examinees be given each pretest item (Ren et al., 2017; van der Linden & Ren, 2015).

The abilities used for assigning the examinees to items are unknown and need to be estimated from an operational test. The estimates can, for example, be derived using the Expected A Posteriori (EAP) method, producing ability estimates with good properties (Bock & Mislevy, 1982). Once the examinee’s responses to the pretest items are available, the pretest item parameters are estimated via maximum likelihood, treating the abilities as fixed. This ignores the uncertainty in the ability estimates, which is one of the factors investigated in the present simulation study. For an approach to handle the uncertainty in the abilities while optimizing the calibration design, see Bjermo et al. (2025).

The item response theory (IRT) models that are being used to describe the relationship between the items and latent abilities depend on the model parameters in a nonlinear way. This means that the optimal allocation design is dependent on the unknown item parameters that are supposed to be estimated, see for example, Atkinsson et al. (2007). The item parameters, therefore, need to be either pre-estimated or given some values by expert guessing; for references justifying the latter see Berger et al. (2019). We explore the effect of pre-estimation in the simulation study by comparing the precision of estimates when the optimal design is derived assuming the true parameter values known to when it is based on pre-estimated values. The SweSAT consists of multiple-choice items and we fit the three-parameter logistic (3PL) IRT model. We note that Berger et al. (2019) investigated and compared by a simulation study calibration designs which can be used when examinees take the test simultaneously in parallel. Their designs are not based on optimality criteria and they assume the Rasch model for the pretest items. They investigated the impact of item-parameter pre-estimation.

The article is organized as follows. We start with describing the 3PL model and the proposed optimal allocation method and give some background about the SweSAT. Next, the details about the setup of the empirically based simulation study, and its different scenarios, are specified along with the definitions of the measures and evaluation metrics used to compare the optimal and random designs. Then the results from the simulation study are presented, and the paper ends with a discussion about the results.

### The 3PL Model

The test items considered here are all multiple-choice questions that have dichotomous outcomes; a response is either correct or incorrect. Therefore, a 3PL model (see e.g., Lord, 1980) is used to model how the probability of a correct response depends on examinee ability. The 3PL model has 3 item parameters $\beta ={\left(a,b,c\right)}^{⊤}$ ; the *a*-parameter is related to item discrimination, *b* reflects item difficulty and *c* is the so-called “guessing” parameter. For the 3PL model the probability that an examinee with ability *θ*_*j*_ correctly responds to item *i* with parameters $\beta_{i}={\left(a_{i},b_{i},c_{i}\right)}^{⊤}$ , is

(1) $$p_{i}\left(\theta_{j}\right)=p_{i}\left(\theta_{j}|a_{i},b_{i},c_{i}\right)=c_{i}+\frac{1-c_{i}}{1+e^{-a_{i}\left(\theta_{j}-b_{i}\right)}}.$$

The 3PL model is a generalized nonlinear model (GNLM) with logit link $\eta_{i}\left(\theta \right)=\log\left(\frac{p_{i}\left(\theta \right)}{1-p_{i}\left(\theta \right)}\right)=\log\left(\frac{c_{i}+\exp\left(-a_{i}\left(\theta -b_{i}\right)\right)}{1-c_{i}}\right)$ , where *p*_*i*_ is the probability defined in equation (1). The link is differentiable in *β*_*i*_.

**Figure 1.**
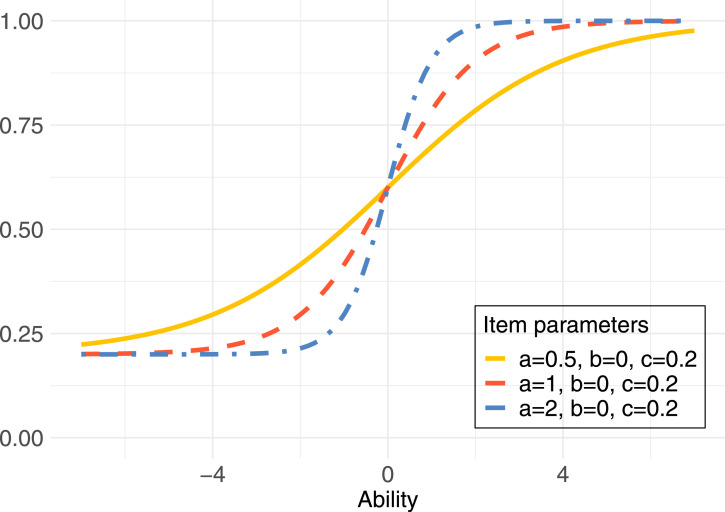
Item response curves with varying discrimination levels

The graph of the probability to correctly respond to an item as a function of examinee ability *θ* illustrates the item characteristics and is often referred to as the *Item Characteristic Curve (ICC)* or item response curve. Figure 1 displays three item response curves with different levels of item discrimination.

### Optimal Design Allocation

An optimal design of an experiment determines the experimental conditions in a way that the estimation is optimal with respect to some criterion, see for example, Silvey (1980). Methods of optimal experimental design are used here to derive an optimal allocation strategy for item calibration. The optimal design allocation provides rules for matching pretest items to examinees based on examinee ability, such that the precision of item-parameter estimates is optimized.

Using an optimal design allocation, the item parameters in the 3PL model will be estimated with better precision compared to a non-optimal design, at least in theory. Since the item-parameter estimates are correlated (Baker & Kim, 2004), we do not focus on the precision of the three model parameters separately. Rather we use here the so-called D-optimality criterion (Atkinsson et al., 2007) which takes the correlation of the parameters into account. This criterion minimizes the determinant of the inverse information matrix (asymptotically equivalent to the covariance matrix) of the item-parameter estimators. This determinant is proportional to the volume of a confidence ellipsoid for the three model parameters.

A standard (unrestricted) optimal design specifies a number of ability levels that would be optimal to sample from. For example, a design where equal proportions of the examinees are divided between two specific *θ* points would be an optimal design under the 2PL model. Such an unrestricted design is feasible if there are no restrictions on the availability of examinees with certain abilities. However, in a real test situation, it is not realistic to be able to choose an examinee with the exact ability needed. Instead, Ul Hassan and Miller (2019) proposed a restricted design that is more reasonable to attain in practice. In the restricted design, ability intervals are instead specified, as opposed to ability points for the unrestricted optimal design.

While Ul Hassan and Miller (2019) have exemplified the restricted design approach for the 2PL model, it is valid even for other IRT models including the 3PL model; the latter model has been considered by Ul Hassan and Miller (2021).

Let *g* be a continuous density on Θ = R which describes the abilities of the examinees; we assume in this article that the examinees have standard normal distributed abilities and *g* is the *N* (0, 1)-density. A restricted design is described by sub-densities *h*_1_, *h*_2_, *…*, *h*_*n*_ ≥ 0 for each item in the test, where $\sum_{i=1}^{n}h_{i}\left(\theta \right)=g\left(\theta \right)$ for all *θ* ∈ Θ. Each non-overlapping *h*_*i*_ represents the part of the examinee population calibrating item *i*.

Writing an expression for the information matrix in a GNLM (see Section 5 of Seber & Wild, 1989) in the notation of Ul Hassan and Miller (2019, 2021), the standardized information matrix of the item parameters *β* = (*β*_1_, *…*, *β*_*n*_) is *M*(*h*) = diag (*M*_1_ (*h*_1_), …, *M*_*n*_ (*h*_*n*_)) with

(2) $$M_{i}\left(h_{i}\right)=\int_{\Theta }p_{i}\left(\theta \right)\left(1-p_{i}\left(\theta \right)\right)\left(\frac{\partial \eta_{i}\left(\theta \right)}{\partial \beta_{i}}\right){\left(\frac{\partial \eta_{i}\left(\theta \right)}{\partial \beta_{i}}\right)}^{T}h_{i}\left(\theta \right)d\theta .$$

Here, the density *h* summarizing the sub-densities *h*_*i*_ describes the allocation rule saying which pretest item 1, *…*, *n* should be given to examinees with a specific ability *θ* (formally, it is defined as *h* (*θ*, *i*) = *h*_*i*_(*θ*) and is a density on the product space Θ × {1, *…*, *n*}). Also, *∂η*_*i*_(*θ*)/*∂β*_*i*_ is the derivative of the logit link described in Section The 3PL Model with respect to *β*_*i*_.

To obtain an optimal design, an appropriate convex function Ψ of *M*(*h*) needs to be optimized; a design h* is Ψ-optimal if h* = arg min_*h*_Ψ(*M*(*h*)).

The considered model is not linear in this case; it means that *M*(*h*) is dependent on the item parameters *β*_*i*_. Therefore, some initial values must be assigned to the item parameters *β*_*i*_. Initial values can be obtained by a guess from an expert or some pre-estimation using a small sample of examinees. The optimal design h* is said to be a locally optimal restricted design (Atkinsson et al., 2007; Ul Hassan & Miller, 2019). A sample of 30 examinees was enough for pre-estimation of item parameters in the situation considered by He and Chen (2020).

As mentioned above, the D-optimality criterion is used which is computed by minimizing

(3) $$\Psi \left\{M\left(h\right)\right\}=-\log|M\left(h\right)|=-\left(\overset{n}{\underset{i=1}{\sum }}\log\left(\left|M_{i}\left(h_{i}\right)\right|\right)\right).$$

**Figure 2.**
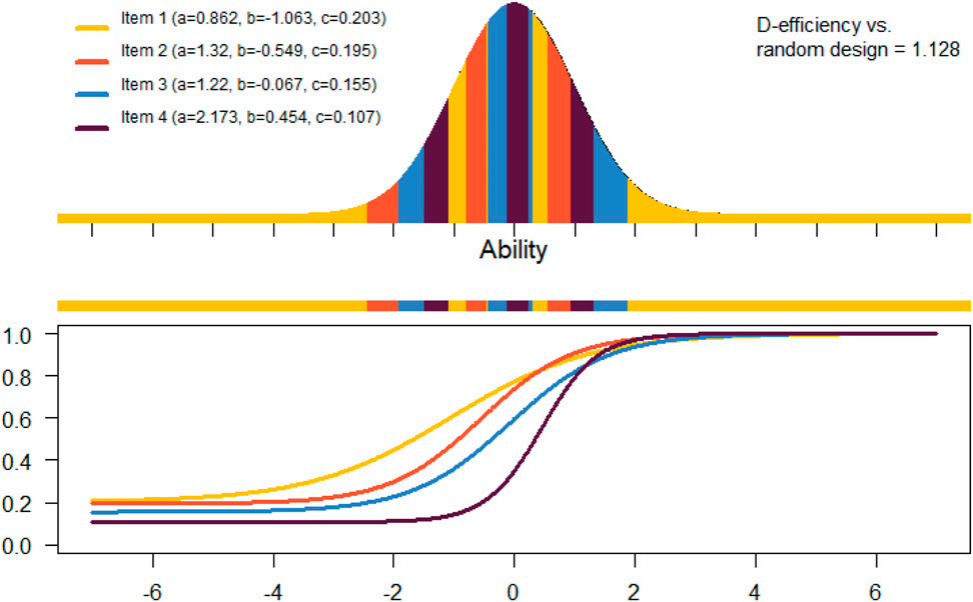
Calibration of 4 items under the 3PL model. Upper panel: The different colors of the normal distribution depict the ability level intervals of the locally D-optimal design for these items. Lower panel: Assumed item response curves for the four pretest items

Ul Hassan and Miller (2019) derived a new equivalence theorem for calibration of multiple items which is able to identify if a design is D-optimal. To find an optimal design, an exchange algorithm can be used (Ul Hassan & Miller, 2021) which is implemented in the R-package optical (Ul Hassan & Miller, 2023). An assumption in the algorithm is that the pretest items are arranged in blocks and that each examinee can be given one pretest item in each block. If, for example, four items are given, the exchange algorithm will find the optimal design for those specific items. The design defines which item an examinee with a certain ability will be given in a calibration situation, see Figure 2 for an example of a produced design. For example, this design recommends that an examinee should receive Item 3 who has, based on the operational items, an ability in one of the four ability intervals: [−1.92, −1.49], [−0.43, −0.13], [0.23, 0.31], [1.32, 1.88]. For this example, the theoretical D-efficiency of this D-optimal design compared to a design which randomly allocates the items to the examinees is 1.128. This means that the random design needs 12.8% more examinees to attain similar information about the parameters compared to the optimal design, see Ul Hassan and Miller (2021).

### The Swedish Scholastic Aptitude Test (SweSAT)

SweSAT is a standardized test used for admission to higher education in Sweden (Umeå University, 2021). The test is given twice a year to around 40,000 examinees. It consists of two main sections—a quantitative and a verbal section. The test is a paper and pencil-based test with a total of five parts; two quantitative parts and two verbal parts, and one try-out/calibration part used for testing the performance of new items. Every part consists of 40 items, all identical across all test locations except for the try-out parts which could differ between test locations. The total score is put on a scale from 0 to 2 and is equated between tests to be comparable.

In this study, we use response data from the quantitative part of the second test round of 2018 and estimate the item parameters which are used as a starting point in our simulation study. The test parts consist of multiple-choice questions with dichotomous outcomes. The 3PL model is often appropriate for analyzing such items and by comparing goodness-of-fit tests, we concluded that the 3PL model had the best fit also in these particular cases.

## Methods

### Calibration of New Items

The method we propose here for calibrating new items can be performed under a separate offline setup or an integrated online setup. For both setups, the estimated ability is used to determine which examinee will be given which pretest item. The items used for estimating the ability are called the operational items, or the operational test. SweSAT uses a separate offline calibration, as described in Section The Swedish Scholastic Aptitude Test (SweSAT). For an integrated setup, the calibration is usually at the end of the test since the estimated abilities have better precision when the estimation can be based on more operational items. In this paper, we are using item parameters that are estimated from the SweSAT. We divide the items into an operational and calibration part, consisting of the same number of items. Which examinee that should be given which pretest item is either determined randomly (random design) or according to the restricted optimal design defined in Section Optimal design allocation. The optimal design should theoretically produce estimates with lowest standard error of the parameters in the item-parameter vector *β*_*i*_ in the 3PL IRT model (Equation (1)).

### Block Design

An assumption in the optical exchange algorithm (Ul Hassan & Miller, 2021) is that an examinee can be given at most one pretest item out of a block of a low number of pretest items. We assume therefore that the pretest items are divided into *l* blocks consisting of *m* items each. The optical algorithm is then run separately on each block, assigning every examinee one item per block. Since the design is locally optimal, the pretest item parameters will need to have an initial value determined, for example, based on a pre-estimation using a small number of examinees.

Every examinee will be given *l* pretest items in addition to the operational items. Which item from each block that should be assigned to the examinees is decided by the estimate of the ability of the examinees, which is estimated based on their responses to the operational test.

Which pretest items will be assigned to which block is decided by the difficulty parameter of the items, either estimated from the full SweSAT data or pre-estimated from a smaller number of examinees. The items could be assigned to the blocks in many different ways. A main idea of design optimization is however that we aim to target the difficulty of the items with the right abilities of the examinees. Therefore, in this paper we choose to assign items to blocks such that each block should have a mix of items with different difficulty levels. We assigned the easiest item to Block 1, the second easiest to Block 2, etc. The *l*-th easiest item will be assigned to block *l*. Then the *l* + 1 easiest item is assigned to Block 1, the *l* + 2 easiest to Block 2 etc. This procedure is continued until all pretest items are assigned to one of the *l* blocks. Table 1 shows an example of a block. This way of assigning the items to the blocks ensures a spread in difficulty between all items within a block. If there would be items with similar difficulty within a block, we would not expect large possibilities to increase efficiency with choosing an optimal design. For example, if we would use four items with *a*- and *c*-parameter like in Table 1 and *b*-parameter equal to −0.306 (the average of the *b*-parameters in Table 1), the D-efficiency would be 1.094, that is, the information gain compared to the random design would be 9.4% instead of 12.8%.

**Table 1.** One Block of Pre-estimated Parameters

	*a*	*b*	*c*
1	0.862	−1.063	0.203
2	1.320	−0.549	0.195
3	1.220	−0.067	0.155
4	2.173	0.454	0.107

### Simulation Setup - 4 Cases

**Figure 3.**
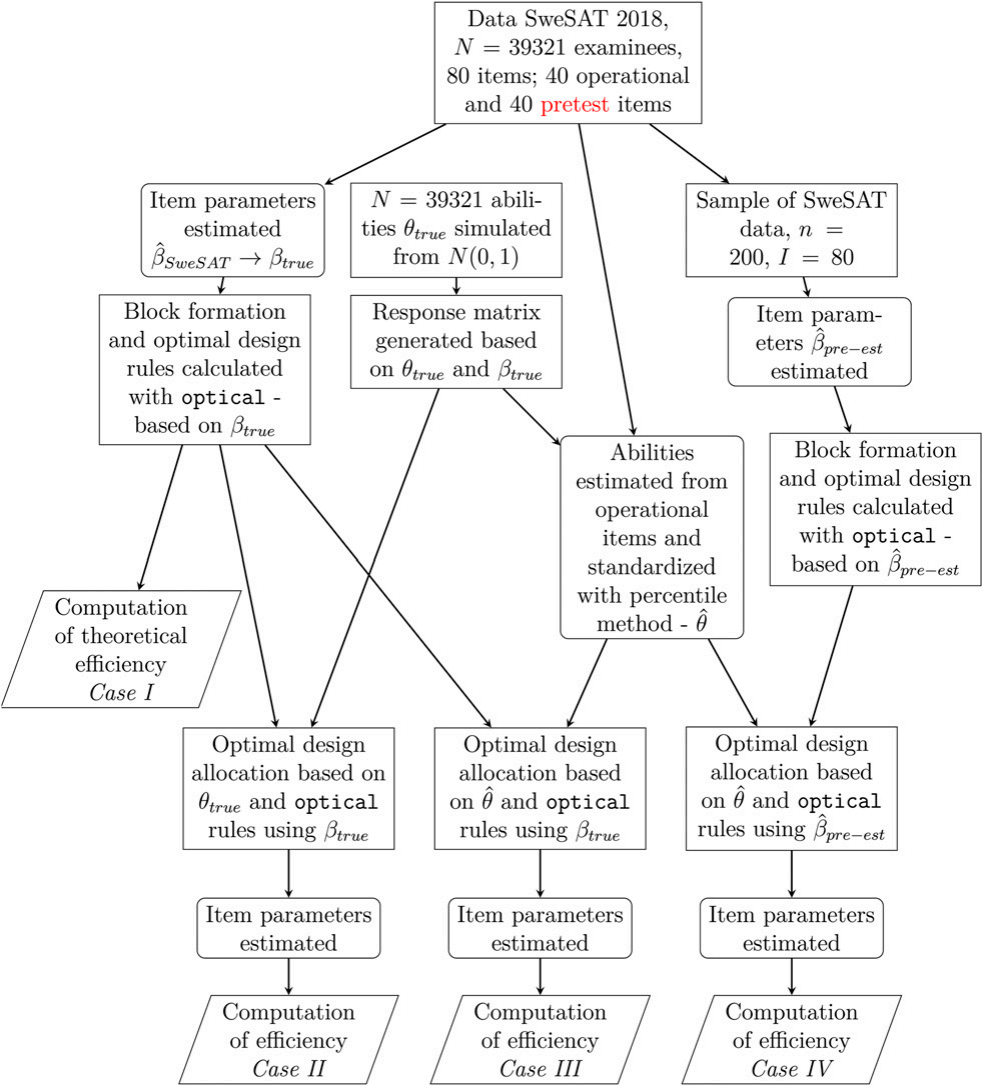
Outline of the simulation studies. Data and design generation is marked with rectangles, estimation of abilities or item parameters is marked as rectangles with rounded corners, and computations for result generation are marked as parallelograms

The proposed method will be evaluated using simulation studies divided into four separate scenarios. An outline of the elements in the simulations under the 4 cases is illustrated in Figure 3. The different cases range from a purely theoretical case to a case replicating a real calibration setting as closely as possible. There are two intermediate steps, where we relax one factor at time, aiming to isolate the influence of each.

The simulation study compares an optimal design allocation to a random allocation of pretest items to examinees, in terms of precision of the estimated item parameters. The simulations are run *S* = 2,000 times. Let the estimated parameter vector for item *i*, *i* = 1, *…*, *I*, in simulation run *s*, *s* = 1, *…*, *S*, when design *d*, *d* = *O* (*Optimal*), *R* (*Random*), is used be

(4) $${\hat{\beta }}_{i,s}^{\left(d\right)}={\left({\hat{a}}_{i,s}^{\left(d\right)},{\hat{b}}_{i,s}^{\left(d\right)},{\hat{c}}_{i,s}^{\left(d\right)}\right)}^{⊤}$$

and the true parameter vector be $\beta_{i}={\left(a_{i},b_{i},c_{i}\right)}^{⊤}$ .

We use the SweSAT data as a starting point with the aim to replicate the SweSAT test and calibration setting in the simulations. The total number of items of the quantitative part of the SweSAT is 80, 39,321 examinees took the test and the 39321 × 80 response matrix is used to estimate the item parameters for the 80 items. This is done via marginal maximum likelihood estimation (MMLE) assuming a normal distribution of the examinee population (Bock & Aitkin, 1981). These MMLE item-parameter estimates are then utilized as the true item parameters *β*_*true*_, when generating a response matrix in each simulation iteration via the 3PL model. Using the same number of examinees as in the SweSAT, 39,321 abilities are drawn from the *N* (0, 1) distribution. These will function as the true abilities of the examinees, *θ*_*true*_, and are also used when generating the simulated response matrices (together with the “true” item parameters *β*_*true*_).

We decided to let the operational and calibration part consist of 40 items each, since each part in SweSAT consists of 40 items. The calibration part will be divided into *l* = 10 blocks with *m* = 4 items per block. This means that every examinee will be given 10 pretest items. Which items each examinee is given is determined by the optimal design on the basis of their EAP ability estimate. This optimal design is to be compared to the random design where 10 item are randomly allocated to every examinee. This means that the sample size per pretest item is about 39321/4 ≈ 9800 for the random design. The 80 true items are randomly divided into an operational part and a calibration part, both of length 40. Responses are simulated by generating draws from the binomial distribution *Bin*(1; *p*_*ij*_ (*θ*_*j*_|*β*_*i*_)) where *p*_*ij*_ are defined as in equation (1). The R-package mirt (Chalmers et al., 2023) was used for both the parameter estimation and generation of response matrices.

#### *Case I* - Theoretical

The first step is to form the blocks of items, so that the optimal design allocation rules can be derived through optical. The blocks are constructed based on the true difficulty parameters *b*_*i*_. The pretest items are sorted from easiest to hardest, and the blocks are created as described in 2.2. The optical algorithm is then run for all blocks, creating the optimal design rules for each block and calculating the theoretical design efficiency. This case does not involve any data generation or parameter estimation once the true parameters *β*_*true*_ are generated, just theoretical calculations of the efficiency of the optimal design allocation compared to a random allocation.

#### *Case II* - True Abilities

For this case, as well as for the two following cases, we generate response matrices based on *θ*_*true*_ and *β*_*i*_ = *β*_*true*_. The optimal design rules derived with optical using *β*_*true*_ are used to assign pretest items to examinees, based on examinee ability, one item from each block. Here, we use the true abilities *θ*_*true*_ also for design allocation. Every examinee is now assigned responses for these items, 10 pretest items each (the non-pretest items will be set to missing in the generated response matrix) and the pretest item parameters are estimated. These item estimates are the optimal estimates ${\hat{\beta }}_{i,s}^{\left(O\right)}$ .

For the random design every examinee is randomly assigned responses for 10 pretest items (and missing values for the rest of the items). The item parameters for the pretest items are estimated using these 10 responses for every respondent, yielding the random design estimates of the item parameters ${\hat{\beta }}_{i,s}^{\left(R\right)}$ .

Design efficiencies are then calculated using the item-parameter estimates of the two designs over the *S* = 2000 simulation runs.

#### *Case III* - Estimated Abilities and True Parameters

In a real calibration situation, examinee abilities are not known and need to be estimated. We use the operational part of the response matrix to first estimate the ability of each examinee ${\hat{\theta }}_{i}^{\left(0\right)}$ ; for details, see Section Estimation of abilities. The optimal design rules derived with optical using *β*_*true*_ are then again used to assign pretest items to examinees, but this time based on the estimated examinee ability. The efficiencies of the resulting item-parameter estimates compared to the random allocation are calculated over the *S* = 2000 simulation runs.

#### *Case IV* - Estimated Abilities and Pre-estimated Parameters

To come even closer to a realistic setup, we relax also the fact that the item parameters used in the design generation are known. Instead, we assume that a small pre-calibration study was done with 200 examinees to obtain initial information about all item parameters which can be used for design generation of the following calibration with *N* = 39321 examinees. Therefore, we simulate 200 responses and pre-estimate values for the pretest items ${\hat{\beta }}_{\mathrm{pre-est}}$ . We use Bayesian estimation using Rstan (Stan Development Team, 2025) to avoid the severe bias that otherwise can appear in the item-parameter estimates when using marginal maximum likelihood estimation (MMLE) for a small number of examinees. The reason for only letting 200 responses pre-estimate the pretest items is that, in a real test situation, you would like to keep the costs down. The pre-estimates are then used for block formation and derivation of the optimal allocation rules through optical. The optical allocation rules that dictate which items the examinees will calibrate are now dependent on their estimated abilities ${\hat{\theta }}_{i}^{\left(0\right)}$ . Item-parameter estimates from the optimal design allocation under this setup are compared to the parameter estimates from the random design, again averaged over the *S* = 2000 simulation runs.

#### Estimation of Parameters

In *Case III* and *IV*, when pretest item parameters are estimated, the ability estimates will be treated as the true abilities. The pretest item parameters are then estimated by maximum likelihood for known fixed abilities, as for regular logistic regression with a non-latent covariate. This approach is similar to the so-called Method A (Stocking, 1988), in the sense that abilities are estimated separately in a first step and treated as fixed in the subsequent step, when the item parameters are estimated. However, Method A uses as a first step separate standard maximum likelihood estimation, via for example, Newton-Raphson maximization, of the ability of each examinee, given the operational item parameters and responses. In our simulations we use instead the EAP ability estimates obtained through MMLE in the first step, which has several advantages over ML for CAT (Bock & Mislevy, 1982). If the ability is not treated as fix, the EM-algorithm used for estimating the item parameters will assume that the examinees abilities follow a standard normal distribution. Since the examinees given a certain pretest item are purposely selected when the optimal design allocation is used, their abilities do not follow the assumed distribution, and the estimates would be biased.

Ban et al. (2001) evaluated Method A among other different estimation methods in the setting of online CAT and concluded that the Multiple Expectation-Maximisation (MEM) performed the best, while Method A was associated with the highest error. Yet, for some models and especially with larger sample sizes, Method A was shown to perform well (Chen et al., 2012, 2017). Chen and Wang (2016) develop and assess an improved version of Method A for multidimensional CAT. Another variant is the adapted methods proposed in He et al. (2017). For our purposes, the possibility to set the abilities according to the distribution used to derive the optimal allocation is essential. Since our method ignores the estimation error of the ability estimates, the estimates of the pretest items will be inaccurate to some extent. A comparison between the simulation *Case II* and *III* provide some insight about this difference.

#### Estimation of Abilities

**Figure 4.**
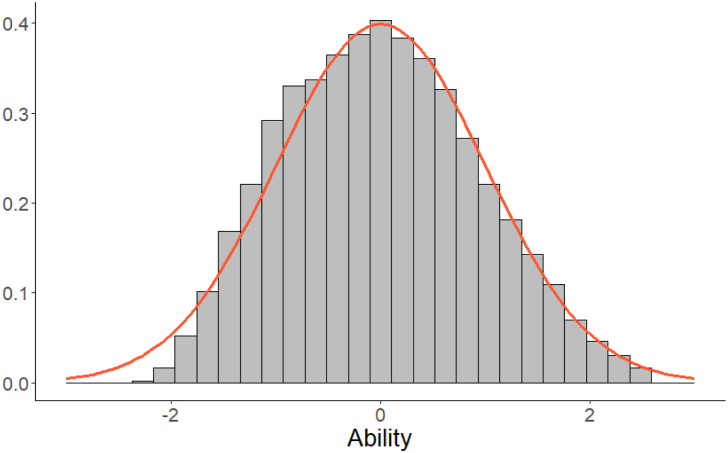
Histogram of estimated abilities ${\hat{\theta }}_{i}^{\left(0\right)}$ in one simulation run and standard normal density as comparison

In *Case III* and *Case IV*, the abilities of the examinees are estimated. Based on the results from the operational part of the test, the ability of each examinee is estimated using the EAP method. Unfortunately, despite that the true abilities are known to be *N* (0, 1) in our simulation studies, the EAP-estimates have a non-normal distribution. The negative abilities are somewhat shrunken towards 0, see Figure 4, which is due to the asymmetry of the 3PL model.

Since the optimal design method assumes a standard normally distributed population, we transform the abilities ${\hat{\theta }}_{i}^{\left(0\right)}$ with the percentile method to values ${\hat{\theta }}_{i}$ distributed as *N* (0, 1), see for example, Kolen and Brennan (2014), Section 9.5.2. The method ranks the estimated abilities ${\hat{\theta }}_{i}^{\left(0\right)}$ and converts the ranks to percentiles by the formula ${\hat{\theta }}_{i}=\Phi^{-1}\left(\left(\text{rank}-0.5\right)/N\right)$ , where *N* is the number of estimated abilities and Φ^−1^ is the inverse cumulative distribution function of the standard normal distribution. By this, the estimated abilities have z-values of the standard normal distribution.

The percentile-transformed estimates ${\hat{\theta }}_{i}$ are then used to determine the pretest item allocation for the optimal design.

### Definitions of Measures

In the following section, several summary measures that are used to evaluate the designs in the simulation studies are presented. For the sake of clarity in the presentation, the index *i* will be temporarily dropped from the formulas in the following section. Note that each measure is defined per item, although the index *i* is not explicitly written out.

#### Error Matrix

The empirical error matrix can be viewed as a multivariate version of the mean squared error of an estimator. When design *d*, *d* = *O*, *R*, is used, it is computed as

(5) $$Q\left({\hat{\beta }}^{\left(d\right)};\beta \right)=\frac{1}{S}\overset{S}{\underset{s=1}{\sum }}\left({\hat{\beta }}_{s}^{\left(d\right)}-\beta \right){\left({\hat{\beta }}_{s}^{\left(d\right)}-\beta \right)}^{⊤}.$$

Based on this, the empirical D-criterion is defined as

(6) $$D\left({\hat{\beta }}^{\left(d\right)};\beta \right)=\det\;\left[Q\left({\hat{\beta }}^{\left(d\right)};\beta \right)\right].$$

#### MSE

The empirical mean squared error for the parameter estimators $\hat{\beta }$ in relation to the true parameters *β* is defined by the 3 × 1 vector

(7) $$MSE\left({\hat{\beta }}^{\left(d\right)};\beta \right)=\frac{1}{S}\overset{S}{\underset{s=1}{\sum }}{\left.\left({\left({\hat{a}}_{s}^{\left(d\right)}-a\right)}^{2},{\left(b_{s}^{\left(d\right)}-b\right)}^{2},{\left(c_{s}^{\left(d\right)}-c\right)}^{2}\right)\right)}^{T}$$

This is equivalent to the diagonal of the empirical error matrix defined above, that is

(8) $$MSE\left({\hat{\beta }}^{\left(d\right)};\beta \right)=diag\;\left[Q\left({\hat{\beta }}^{\left(d\right)};\beta \right)\right].$$

To summarize the MSE of all three parameters in the model, the average MSE is computed:

(9) $$AMSE\left({\hat{\beta }}^{\left(d\right)};\beta \right)=\left\{MSE\left({\hat{a}}^{\left(d\right)};a\right)+MSE\left({\hat{b}}^{\left(d\right)};b\right)+MSE\left({\hat{c}}^{\left(d\right)};c\right)\right\}/3.$$

This measure is the empirical counterpart of the A-optimality criterion

(10) $$\Psi \left\{M\left(h\right)\right\}=\text{tr}M^{-1}\left(h\right)$$

which is the same as minimizing the average variance (Atkinsson et al., 2007, p. 135).

#### CC Method

Inspired by the Haebara approach for IRT test equating (Kolen & Brennan, 2014), the squared difference between ICCs based on estimated and true parameters, is evaluated for a certain ability level *θ*_*j*_. The empirical characteristic curve difference is

(11) $$d\left(\theta_{j}|{\hat{\beta }}^{\left(d\right)};\beta \right)=\frac{1}{S}\overset{S}{\underset{s=1}{\sum }}{\left\{p\left(\theta_{j}|{\hat{a}}_{s}^{\left(d\right)},{\hat{b}}_{s}^{\left(d\right)},{\hat{c}}_{s}^{\left(d\right)}\right)-p\left(\theta_{j}|a,b,c\right)\right\}}^{2}.$$

To judge the overall difference between estimated and true ICCs, we can integrate this difference over *θ* (area between curves). If we weight according to the distribution of the examinees, the integral corresponds to the sum over the abilities of the *j* = 1, *…*, *N* examinees. This yields the total difference

(12) $$CC\left({\hat{\beta }}^{\left(d\right)};\beta \right)=\overset{N}{\underset{j=1}{\sum }}d\left(\theta_{j}|{\hat{\beta }}^{\left(d\right)};\beta \right).$$

### Evaluation Metrics

To be able to evaluate which of the two designs: *Optimal (O)* or *Random (R)* performs better; several metrics are calculated as ratios of the previously defined measures. These ratios are called (relative) efficiencies; when they exceed 1, the parameters are estimated less precisely with the random design compared to the optimal design. We can interpret the efficiencies in terms of sample size: An efficiency of 1.25 means that 25 % more examinees are needed for the random design compared to the optimal design to achieve a similar precision of the estimates, independent of the actual sample sizes used.

#### Relative Average MSE

The relative average MSE, or relative A-efficiency, is given by the ratio of the empirical AMSE of the random design (*R*) and the optimal design (*O*)

(13) $${RE}_{\text{A}}=\frac{AMSE\left({\hat{\beta }}^{\left(R\right)};\beta \right)}{AMSE\left({\hat{\beta }}^{\left(O\right)};\beta \right)}.$$

#### Relative D-efficiency

The relative D-efficiency is obtained as the ratio of the empirical D-criterion of the random design (*R*) and the optimal design (*O*)

(14) $${RE}_{\text{D}}={\left(\frac{D\left({\hat{\beta }}^{\left(R\right)};\beta \right)}{D\left({\hat{\beta }}^{\left(O\right)};\beta \right)}\right)}^{\frac{1}{3}}.$$

The exponent 1/3 (in general, 1/(number of parameters in the model)), is needed for scaling such that we can interpret it in terms of sample size, see for example, Atkinsson et al. (2007), Section 11.

#### Relative CC-efficiency

The relative CC-efficiency is defined as the ratio of the CC-criterion of the random design (*R*) and the optimal design (*O*)

(15) $${RE}_{\text{CC}}=\frac{CC\left({\hat{\beta }}^{\left(R\right)};\beta \right)}{CC\left({\hat{\beta }}^{\left(O\right)};\beta \right)}.$$

#### Overall Evaluation

All of the previously defined measures are calculated *per item*. For an overall assessment across all 40 items, we take the geometric mean over them. Since efficiencies are relative measures, the geometric mean is more appropriate than the arithmetic mean. For example, if Item 1 has efficiency 0.5, Item 2 has 2, then the random design needs half of the examinees for Item 1 and double the examinees for Item 2 to obtain similar precision as the optimal design. Overall, both designs should then be of the same quality which is reflected by the geometric mean of 1. In contrast, the arithmetic mean would be 1.25 suggesting an advantage of the optimal design. The arithmetic mean would give a too optimistic overall measure; the geometric mean is always smaller than or equal to the arithmetic mean.

## Results

The results are presented as the evaluation metric per item based on averages taken over the *S* = 2,000 simulation runs. The number of runs was chosen to obtain a precision of relative item efficiencies of ±0.05 (we have derived bootstrap 95%-confidence interval for the simulation error in some cases which are approximately of that size but we have not included them in the results later for sake of clarity). This means that values ≥1.05 are significantly larger than 1 given the Monte Carlo precision; and values ≤0.95 are significantly smaller than 1. The average relative efficiency for all pretest items has then a precision of less than ±0.01.

All four simulation cases have been evaluated and the results of the overall evaluations are given in Table 2. Results per item of *Case III* and *Case IV* are presented in more detail in this section. The theoretical efficiencies per item of *Case I* are given in Table A1 in the appendix. The results of *Case II* are very similar to those, and the per item details are therefore omitted.

**Table 2.** Relative Efficiencies RE_D_, RE_CC_, and RE_A_ for Optimal Versus Random Design, Summarized for All of the 40 Items for the Three Measures (Geometric Mean)

	RE_D_	RE_CC_	RE_A_
*Case I*: Theoretical	1.155	1.126	1.426
*Case II*: True abilities	1.155	1.105	1.346
*Case III*: Estimated abilities and true parameters	1.103	1.080	1.198
*Case IV*: Estimated abilities and pre-estimated parameters	1.048	1.048	1.093

Since the D-optimality criterion is the criterion that was used for optimization of the design, the D-efficiency RE_D_ will be in focus when examining the results. Plots to display the correlation between the true item parameters and the relative D-efficiency RE_D_ are given. Corresponding plots of RE_CC_ and RE_A_ can be found in the appendix.

A general result is that the proposed method using the block design and the optical algorithm manages to produce item parameters estimated with better precision in a majority of the cases.

For Case IV (Table 3), we conclude that 6 items have a *RE*_*D*_ significantly lower than 1, 12 items have *RE*_*D*_ not significantly different from 1, and 22 items have *RE*_*D*_ significantly higher than 1. For Case III (Table 4), the corresponding number of items are 4, 7, and 29. Based on the *RE*_*D*_ in Table 3, we can see that 21 items will need 5% or more examinees for the random design to obtain the same precision of the parameter estimates compared to the optimal design. Also, as expected, the three measures RE_D_, RE_CC_, and RE_A_ are correlated; for example, the Pearson correlation between RE_D_ and RE_CC_ is 0.68. As were explained in Section Evaluation metrics, a relative efficiency >1 can be interpreted as the multiplication factor of the pretest item’s sample size in the optimal design that gives the same estimation precision as the random design. Since the item sample size is about 9,800 for each pretest items when the random design is used, a *RE*_*D*_ = 1.169 (item 4, block 1) means that a sample size of 9800/1.169 ≈ 8383 is sufficient for the optimal design to achieve the same precision. When the pretest item parameters are pre-estimated the overall measure of RE_D_ are lower compared to when the item-parameters used are the true ones, as shown in Table 2. This gives us an indication of the magnitude of the efficiency loss due to the uncertainty of the estimation of item parameters. Roughly half of the overall efficiency gain over the random allocation is lost due to the pre-estimation of item parameters. From Table 2, we can calculate the pretest item’s sample size in the optimal design that would give the same estimation precision as the sample size 9,800 when using the random design. For Case IV with *RE*_*D*_ = 1.048, it will be 9800/1.048 ≈ 9351. It means that about 4.8 % or 450 more examinees are needed if the random design is used instead of the optimal design.

**Table 3.** Relative Efficiencies RE_D_, RE_CC_, and RE_A_ for Optimal Versus Random Design and True Item Parameters *a*, *b*, *c*; Per Item. *Case IV*: Optimal Allocation Based On Estimated Abilities and Pre-estimated Parameters

Block	Pos	RE_D_	RE_CC_	RE_A_	*a*	*b*	*c*	Item
1	1	1.007	1.026	1.279	0.799	−1.516	0.071	39
1	2	1.033	1.090	1.079	1.281	−0.466	0.189	8
1	3	1.077	1.035	1.255	0.924	−0.389	0.147	17
1	4	1.169	1.119	1.135	2.385	0.607	0.191	25
2	1	1.029	1.344	1.254	1.803	−0.900	0.310	20
2	2	1.081	1.136	1.138	1.911	−0.376	0.240	11
2	3	0.997	0.951	0.850	2.203	0.315	0.461	26
2	4	1.056	1.016	1.023	1.582	0.694	0.275	19
3	1	0.925	1.041	0.861	1.458	−0.739	0.318	3
3	2	1.117	1.103	1.330	1.399	−0.346	0.150	35
3	3	1.258	1.174	1.337	1.782	−0.108	0.140	2
3	4	1.072	1.003	1.126	1.676	0.513	0.185	29
4	1	0.889	0.922	0.782	1.333	−0.648	0.296	21
4	2	1.031	1.037	1.161	0.876	−0.792	0.016	27
4	3	1.052	1.036	0.999	0.989	−0.170	0.093	38
4	4	1.094	1.032	1.238	1.144	0.510	0.169	40
5	1	0.990	1.037	0.954	0.983	−1.227	0.052	31
5	2	1.049	1.050	1.152	1.334	−0.241	0.187	4
5	3	1.083	1.099	1.136	1.930	0.315	0.266	24
5	4	1.118	1.017	1.195	1.187	0.806	0.137	18
6	1	0.841	0.898	0.735	1.627	−0.184	0.375	1
6	2	1.087	1.075	1.238	1.781	−0.228	0.073	5
6	3	1.105	1.077	1.157	1.426	0.421	0.158	12
6	4	0.917	1.010	0.832	2.238	1.116	0.301	6
7	1	0.999	1.006	1.314	0.775	−1.223	0.010	36
7	2	1.013	1.057	1.052	1.070	−0.428	0.116	37
7	3	1.160	1.130	1.216	2.374	0.208	0.281	23
7	4	1.175	1.138	1.153	1.789	1.169	0.274	30
8	1	0.878	0.864	0.793	1.181	−0.270	0.372	7
8	2	1.075	0.975	0.992	1.652	−0.590	0.166	22
8	3	1.050	1.035	1.281	0.794	−0.454	0.004	14
8	4	1.197	1.136	1.207	0.946	1.250	0.100	13
9	1	0.915	0.919	1.000	0.813	−0.949	0.137	15
9	2	1.198	1.155	1.242	1.369	−0.140	0.210	33
9	3	0.953	0.935	1.193	1.407	0.427	0.358	16
9	4	1.147	1.114	1.116	1.200	0.741	0.096	34
10	1	1.054	1.136	1.237	1.214	−0.691	0.062	32
10	2	1.006	1.030	0.968	1.471	0.196	0.225	28
10	3	1.134	1.074	1.245	1.256	0.157	0.138	9
10	4	1.037	1.026	0.998	1.687	1.147	0.207	10

**Table 4.** Relative Efficiencies RE_D_, RE_CC_, and RE_A_ for Optimal Versus Random Design and True Item Parameters *a*, *b*, *c*; Per Item. *Case III*: Optimal Design Allocation Based On Estimated Abilities and True Item Parameters

Block	Pos	RE_D_	RE_CC_	RE_A_	*a*	*b*	*c*	Item
1	1	0.814	0.808	0.693	0.799	−1.516	0.071	39
1	2	1.073	1.079	1.017	1.281	−0.466	0.189	8
1	3	1.090	1.028	1.245	0.989	−0.170	0.093	38
1	4	1.187	1.081	1.443	1.144	0.510	0.169	40
2	1	0.878	0.857	0.768	0.983	−1.227	0.052	31
2	2	1.038	0.931	1.511	0.794	−0.454	0.004	14
2	3	1.154	1.119	1.243	1.369	−0.140	0.210	33
2	4	1.250	1.162	1.295	1.676	0.513	0.185	29
3	1	1.003	0.995	1.463	0.775	−1.223	0.010	36
3	2	1.095	1.115	1.361	1.070	−0.428	0.116	37
3	3	1.154	1.101	1.224	1.782	−0.108	0.140	2
3	4	1.244	1.160	1.255	2.385	0.607	0.191	25
4	1	0.890	0.918	0.832	0.813	−0.949	0.137	15
4	2	1.034	1.013	1.277	0.924	−0.389	0.147	17
4	3	1.189	1.153	1.340	1.256	0.157	0.138	9
4	4	1.235	1.167	1.395	1.582	0.694	0.275	19
5	1	1.214	1.408	1.468	1.803	−0.900	0.310	20
5	2	1.146	1.149	1.227	1.911	−0.376	0.240	11
5	3	1.124	1.035	1.102	1.471	0.196	0.225	28
5	4	1.116	1.044	1.149	1.200	0.741	0.096	34
6	1	0.902	0.899	1.055	0.876	−0.792	0.016	27
6	2	1.108	1.123	1.266	1.399	−0.346	0.150	35
6	3	1.219	1.107	1.210	2.374	0.208	0.281	23
6	4	1.189	1.070	1.363	1.187	0.806	0.137	18
7	1	0.979	1.148	0.955	1.458	−0.739	0.318	3
7	2	1.128	1.136	1.479	1.181	−0.270	0.372	7
7	3	1.218	1.162	1.223	2.203	0.315	0.461	26
7	4	1.017	0.918	0.951	2.238	1.116	0.301	6
8	1	1.037	1.135	1.148	1.214	−0.691	0.062	32
8	2	1.157	1.145	1.415	1.334	−0.241	0.187	4
8	3	1.199	1.166	1.294	1.930	0.315	0.266	24
8	4	1.114	1.050	1.015	1.687	1.147	0.207	10
9	1	1.045	1.126	1.197	1.333	−0.648	0.296	21
9	2	1.200	1.177	1.370	1.781	−0.228	0.073	5
9	3	1.191	1.171	1.256	1.426	0.421	0.158	12
9	4	1.087	1.040	1.035	1.789	1.169	0.274	30
10	1	1.189	1.327	1.482	1.652	−0.590	0.166	22
10	2	1.166	1.091	1.205	1.627	−0.184	0.375	1
10	3	1.115	1.034	1.169	1.407	0.427	0.358	16
10	4	1.147	1.085	1.227	0.946	1.250	0.100	13

**Figure 5.**
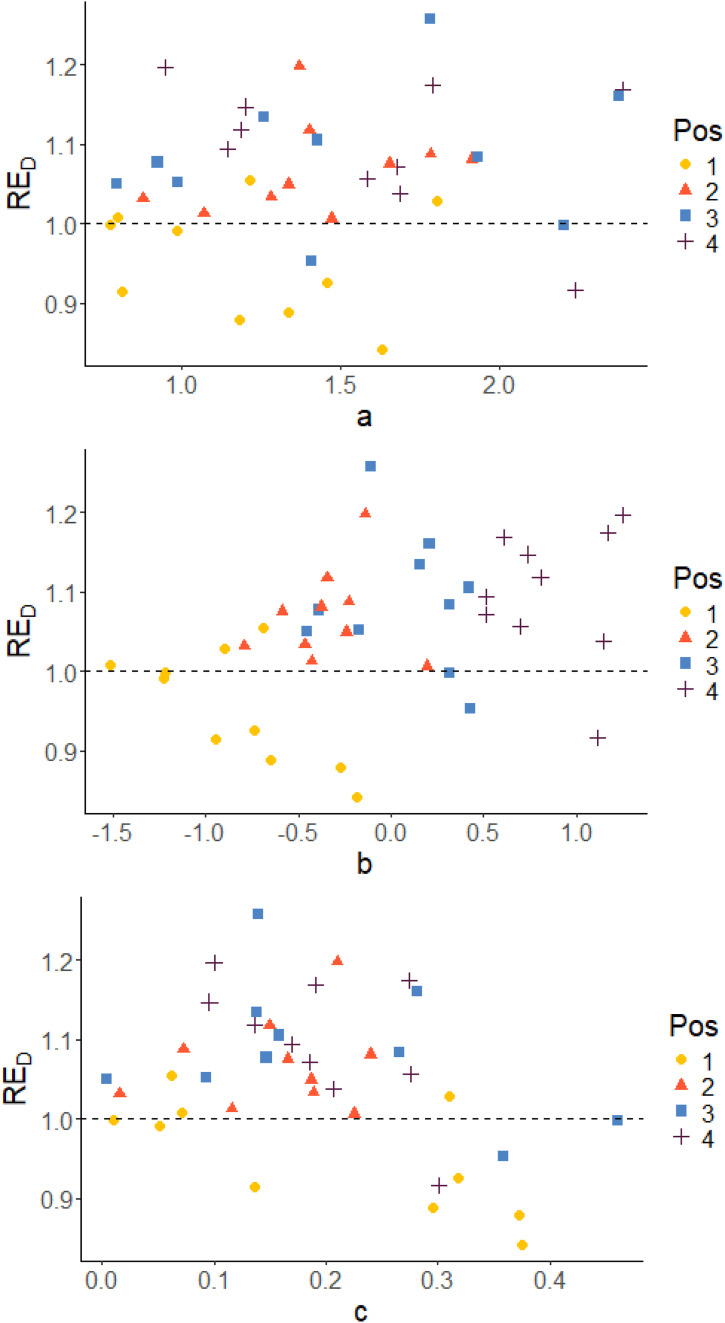
True item parameters plotted against RE_*D*_, grouped on position in the block. *Case IV*: Optimal allocation based on estimated abilities and pre-estimated parameters

**Figure 6.**
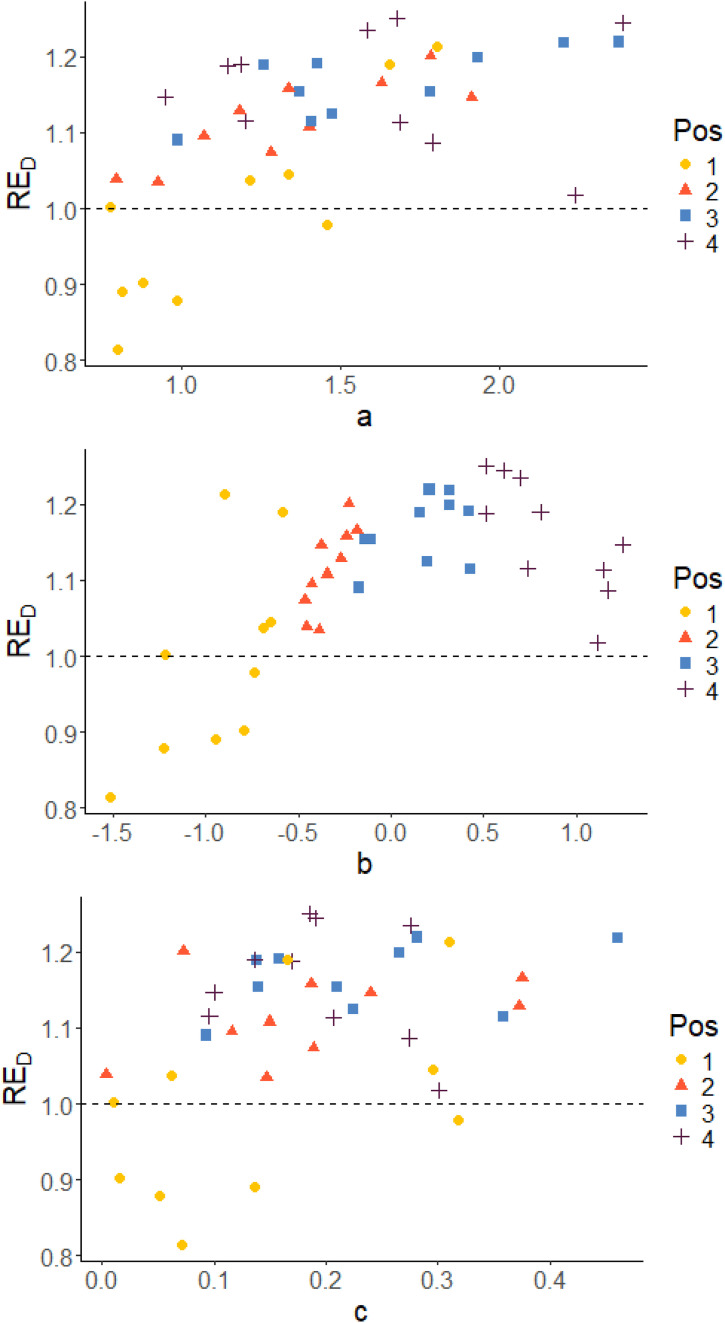
True item parameters plotted against RE_*D*_, grouped on position in the block. *Case III*: optimal design allocation based on estimated abilities and true item parameters

**Figure A1.**
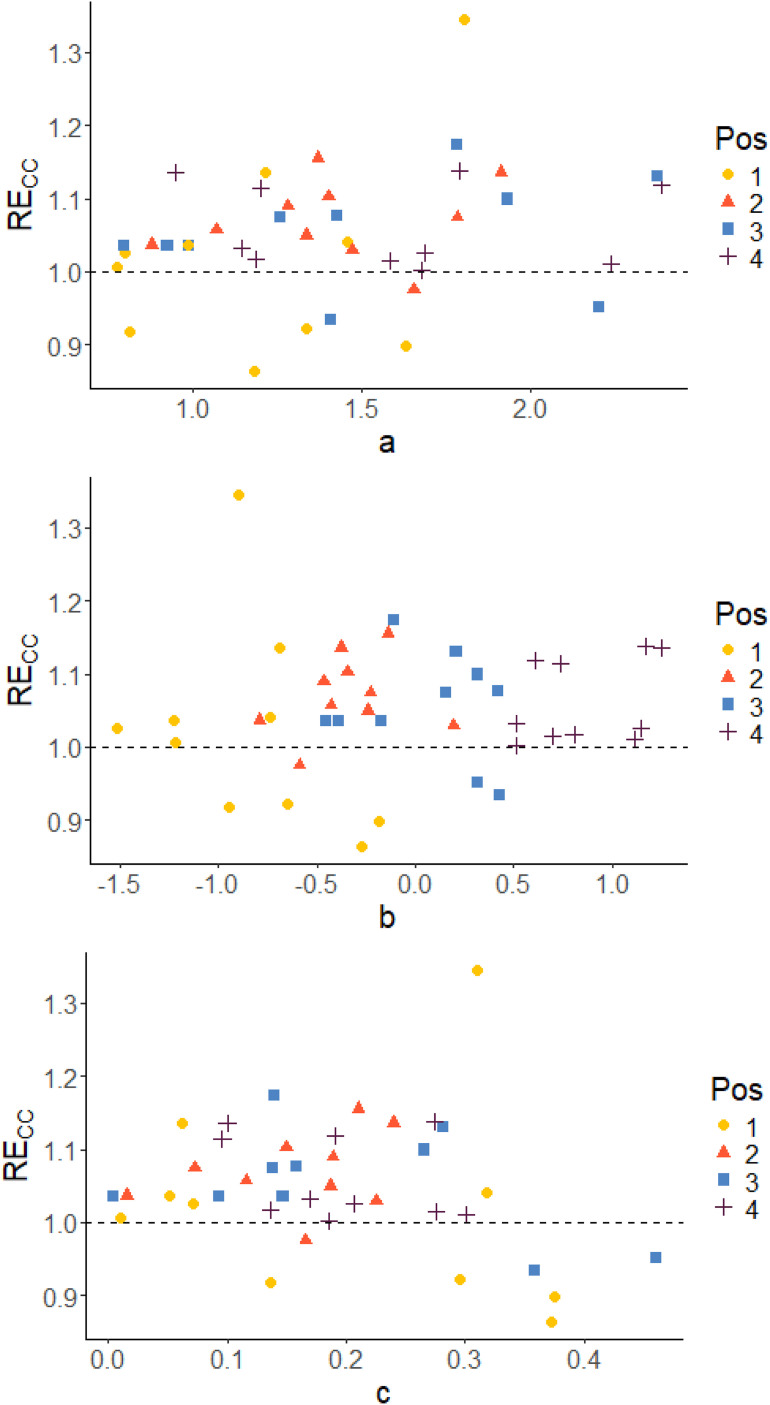
True item parameters plotted against RE_CC_, grouped on position in the block. *Case IV*: Optimal allocation based on estimated abilities and pre-estimated parameters

**Figure A2.**
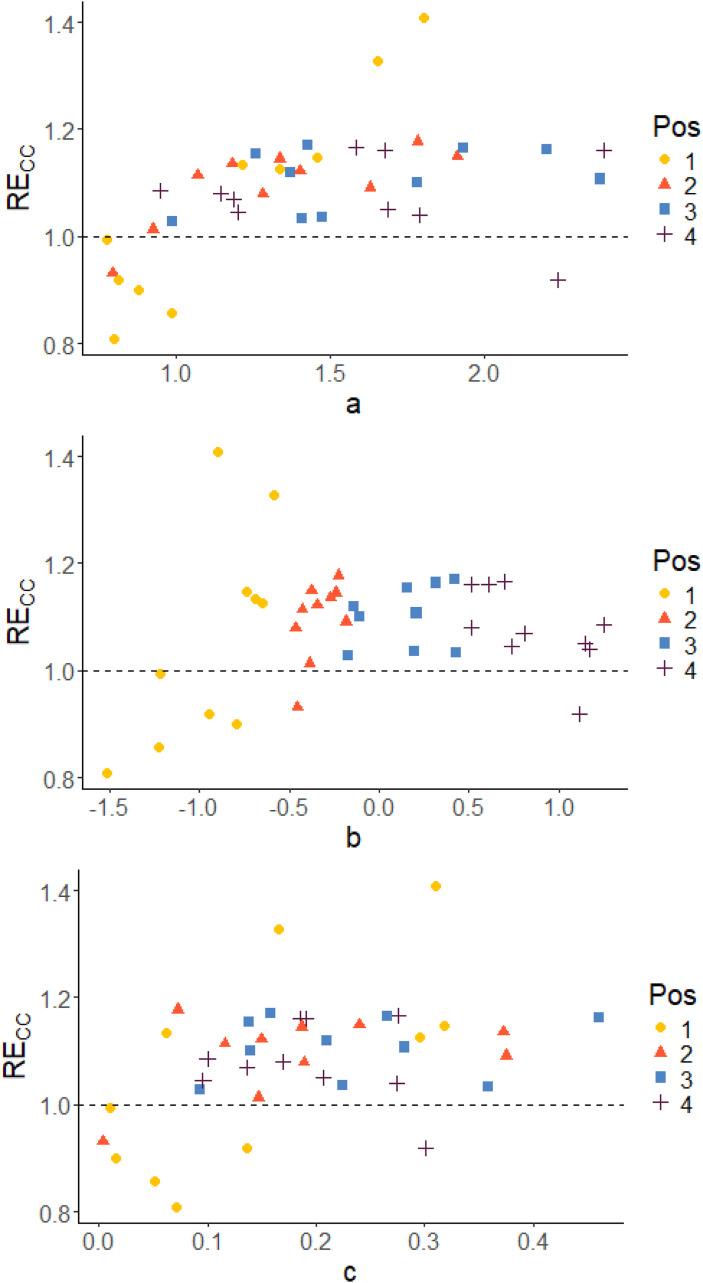
True item parameters plotted against RE_CC_, grouped on position in the block. *Case III*: optimal design allocation based on estimated abilities and true item parameters

**Figure A3.**
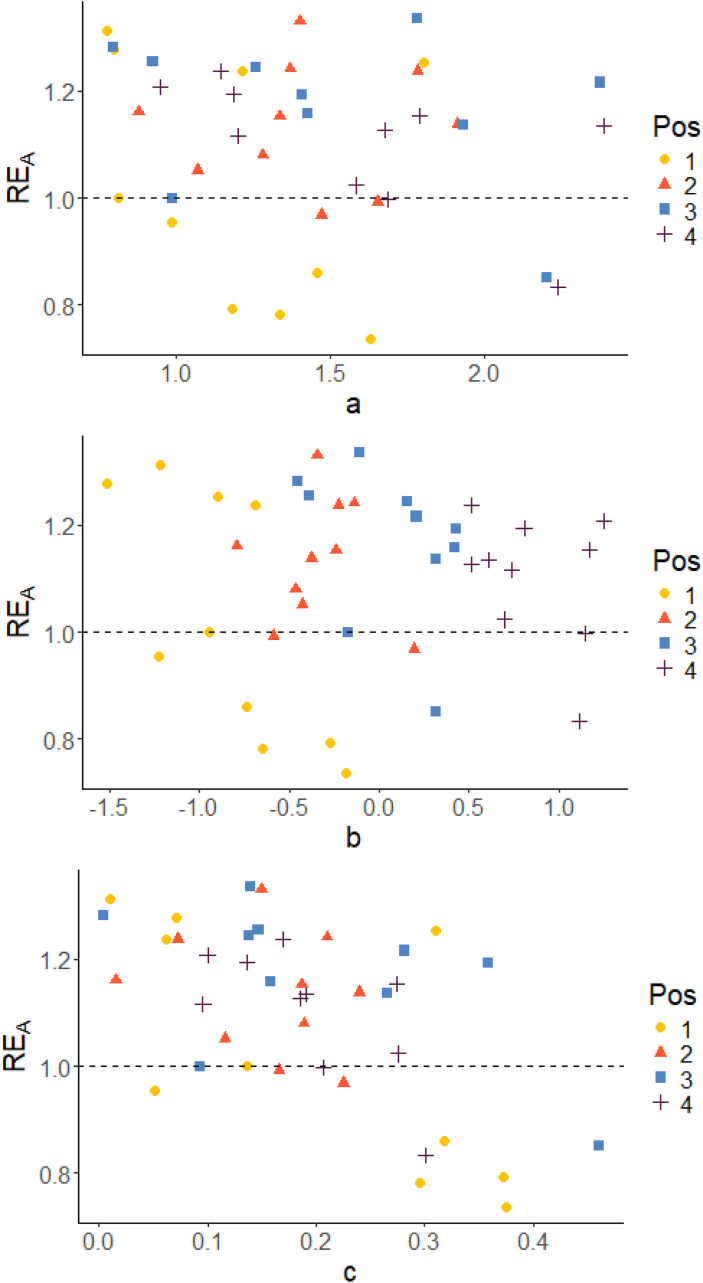
True item parameters plotted against RE_A_, grouped on position in the block. *Case IV*: Optimal allocation based on estimated abilities and pre-estimated parameters

**Figure A4.**
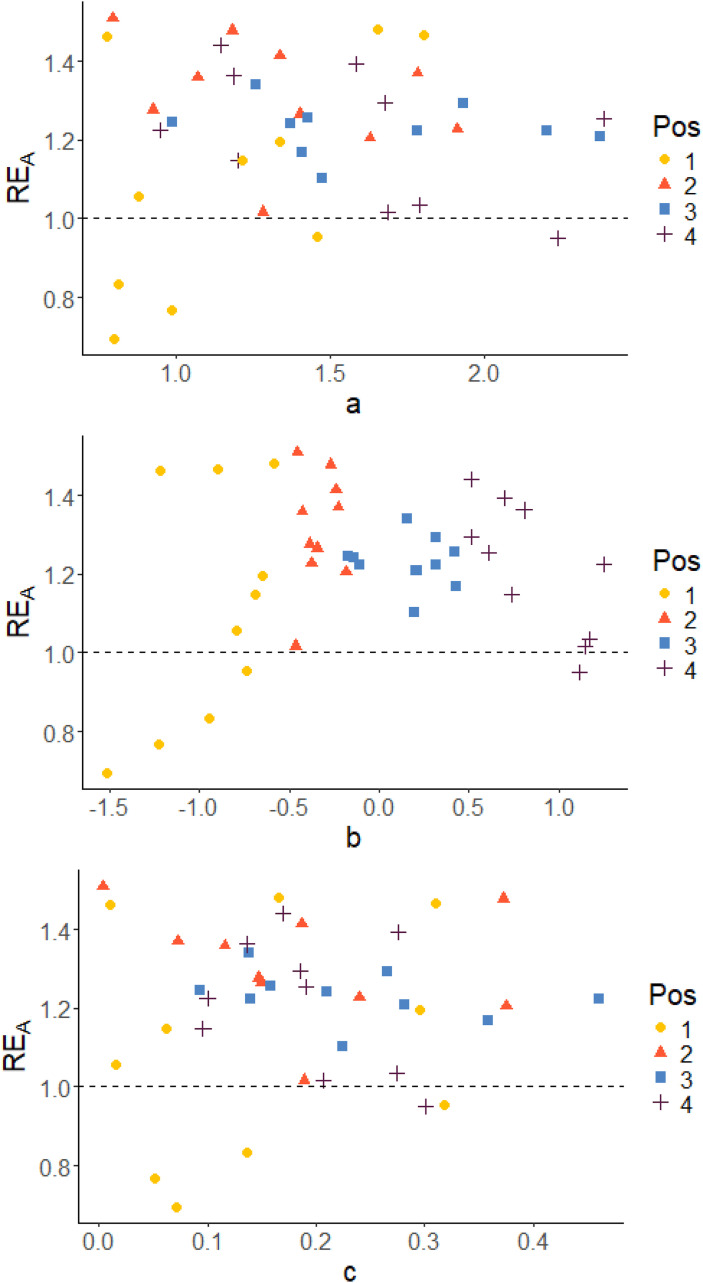
True item parameters plotted against RE_A_, grouped on position in the block. *Case III*: optimal design allocation based on estimated abilities and true item parameters

Figures 5 and 6 show scatter plots of RE_D_ versus true item parameters for *Case IV* with pre-estimated item parameters and *Case III* using true item parameters, respectively. The colors of the dots indicate the position in the blocks. The position in the block is determined by the difficulty of the item (*b*_*i*_-parameter), which means that position is a measure of the relative difficulty in the blocks. In both cases, irrespective of which parameter we examine, it is almost always the easiest items in each block that stands out as being less precisely estimated in terms of D-efficiency RE_D_ (with just a few exceptions). The per item efficiencies generally have higher values for *Case III* compared to *Case IV*, since we do not have any item-parameter uncertainty when the item parameters are assumed known in *Case III*. A similar pattern is observed also with respect to the other two measures, as can be seen in Figures A1 to A4 in the Appendix.

## Discussion

We studied four different simulation scenarios with varying degree of practicality, from entirely theoretical to the most realistic, designed to be as close as possible to the SweSAT setup. This allows us to quantify the influence of different factors (on average) by comparing the overall efficiencies, summarized over all items, between the scenarios. We conclude that there is an efficiency loss at each step, especially between cases *III* and *IV* (pre-estimation of item parameters) but also cases *II* and *III* (estimation of abilities). For the most realistic *Case IV*, the optimal design allocation was still better than the random design allocation, being about 5% more efficient in terms of RE_*D*_ and RE_*CC*_ and almost 10% in terms of RE_*A*_. It means that 5% more examinees are needed for the random design compared to the optimal design to obtain the same precision in the item-parameter estimates.

The results per item show that the optimal design method estimates the pretest items more efficiently compared to the random design in most of the cases. Also, analyzing the block positions, we are able to identify that it is mainly for the easiest items in the blocks the optimal design method is inferior to the random allocation. This pattern can be observed both when the item parameters used in the optimization are pre-estimated (*Case IV*) and when true item parameters were used (*Case III*), see Figures 5 and 6. The reason for that is the asymmetry of the 3PL model, which implies that the *c*-parameters are estimated based on examinees with low abilities. It is more difficult to estimate *c* when the item is easy (low *b*). Since it is more difficult to estimate *b* and *c* for easy items, the optimal design puts more focus on the other items in the block. Their precision can easier be improved leading to an increased overall efficiency even if accepting a decreased efficiency for the easy items. In reality, most of the examinees’ abilities will however lay in *θ* ∈ [−2, 2]. In that span, two items can have similar Item Characteristic Curves even if the item parameters differ.

Since most of the abilities of examinees will lay in the span *θ* ∈ [−2, 2] the consequences for using such item in an item bank might not be that severe (even if the item parameters are less precisely estimated).

As noted, the overall RE_*D*_ (Table 2) is lower when the pretest item parameters are pre-estimated (*Case IV)* compared to when true item parameters are used (*Case III*). For individual items, there can be even larger differences, for example, Item 1 in Table 3 and in 4. This indicates that the effect of putting more focus on the harder items is bigger in the scenario where the pretest item parameters are pre-estimated. But even if the item parameters used in optical are not the true ones, the optimal block design seems to work for a majority of the items. That is, only 6 items have a *RE*_*D*_ significantly lower than 1. When comparing the two different cases it needs however to be considered that the division into the blocks is different since the pre-estimated item parameters will differ from true ones. But since the difference between the overall measures (Table 2) is not so big, it can be concluded that the method functions well when using the pre-estimates that are produced from only 200 examinees. An alternative to pre-estimation is using experts’ guesses. However, Attali et al. (2014); Bejar (1981) suggests that experts often perform poorly when estimating item difficulty. An alternative is to use expert judgmental information (Swaminathan et al., 2003) when constructing priors for Bayesian pre-estimation of item parameters. The item-parameter estimation precision in the 200 examinee pre-estimation example used in this article can then be improved by using expert judgmental information.

It is shown that with the proposed method, most of the items are estimated with higher efficiency and that it is possible to identify when it is not useful. Since it is the easier item in every block that is estimated with less efficiency, it would be beneficial if they can be identified in advance. Even though the exact value of the parameter *b* is not known, it is reasonable to believe that the items can be ranked with respect to difficulty by using, for example, comparative judgments. Attali et al. (2014) showed that judges where able to rank several item in terms difficulty. The items that are probable to be estimated with worse efficiency for the optimal design could then be estimated through a random design for example.

In this paper, we are not taking into account practical issues with assigning items. It could be necessary to put constraints on the pretest items, for example, on items that cannot be simultaneously included in the calibration part. There must also be a mix of content in the pretest items, and they need to be compatible with the operational test items.

In the optimal design approach we are using, it is assumed that the abilities of the examinees are known. In the Cases III and IV of our study, we apply this optimal design for abilities which are estimated. This discrepancy leads to the drop in efficiency observed. However, it could be possible in future research to modify the optimal design approach to include the uncertainty around the ability estimates and to determine optimal designs for the situation of estimated abilities. With these optimal designs, it could be possible to increase efficiency in the case of estimated abilities.

Also, instead of using a pre-estimated point estimate or guess for the pretest item parameters, a prior distribution could be assigned from which an optimal-on-average (also known as Bayesian) design could be derived.

## Appendix

**Table A1.** Theoretical Relative Efficiencies RE_D_, RE_CC_, and RE_A_ for Optimal Versus Random Design and True Item Parameters *a*, *b*, *c*; Per Item. *Case I*: Optical Based On True Item Parameters

Block	Pos	RE_D_	RE_CC_	RE_A_	*a*	*b*	*c*	Item
1	1	1.124	1.043	1.580	0.799	−1.516	0.071	39
1	2	1.103	1.083	1.195	1.281	−0.466	0.189	8
1	3	1.060	1.070	1.283	0.989	−0.170	0.093	38
1	4	1.188	1.199	1.383	1.144	0.510	0.169	40
2	1	1.136	1.067	1.612	0.983	−1.227	0.052	31
2	2	1.076	1.096	1.474	0.794	−0.454	0.004	14
2	3	1.146	1.130	1.252	1.369	−0.140	0.210	33
2	4	1.226	1.248	1.453	1.676	0.513	0.185	29
3	1	1.121	1.052	1.811	0.775	−1.223	0.010	36
3	2	1.082	1.056	1.434	1.070	−0.428	0.116	37
3	3	1.190	1.138	1.187	1.782	−0.108	0.140	2
3	4	1.293	1.329	1.557	2.385	0.607	0.191	25
4	1	1.076	1.012	1.494	0.813	−0.949	0.137	15
4	2	1.052	1.028	1.350	0.924	−0.389	0.147	17
4	3	1.105	1.093	1.208	1.256	0.157	0.138	9
4	4	1.224	1.250	1.499	1.582	0.694	0.275	19
5	1	1.230	1.100	1.574	1.803	−0.900	0.310	20
5	2	1.155	1.196	1.411	1.911	−0.376	0.240	11
5	3	1.104	1.176	1.213	1.471	0.196	0.225	28
5	4	1.108	1.050	1.249	1.200	0.741	0.096	34
6	1	1.149	1.107	1.866	0.876	−0.792	0.016	27
6	2	1.143	1.079	1.314	1.399	−0.346	0.150	35
6	3	1.252	1.254	1.325	2.374	0.208	0.281	23
6	4	1.191	1.169	1.503	1.187	0.806	0.137	18
7	1	1.167	0.974	1.362	1.458	−0.739	0.318	3
7	2	1.099	1.025	1.384	1.181	−0.270	0.372	7
7	3	1.213	1.218	1.351	2.203	0.315	0.461	26
7	4	1.256	1.363	1.785	2.238	1.116	0.301	6
8	1	1.157	1.033	1.619	1.214	−0.691	0.062	32
8	2	1.125	1.044	1.365	1.334	−0.241	0.187	4
8	3	1.187	1.184	1.408	1.930	0.315	0.266	24
8	4	1.261	1.236	1.855	1.687	1.147	0.207	10
9	1	1.109	0.997	1.450	1.333	−0.648	0.296	21
9	2	1.140	1.089	1.201	1.781	−0.228	0.073	5
9	3	1.151	1.165	1.318	1.426	0.421	0.158	12
9	4	1.261	1.295	1.876	1.789	1.169	0.274	30
10	1	1.237	1.156	1.586	1.652	−0.590	0.166	22
10	2	1.146	1.186	1.337	1.627	−0.184	0.375	1
10	3	1.123	1.203	1.207	1.407	0.427	0.358	16
10	4	1.103	1.022	1.198	0.946	1.250	0.100	13
